# Melorheostosis: A Systematic Review of Clinical Manifestations, Diagnostic Challenges, Therapeutic Strategies, and Physiotherapeutic Interventions

**DOI:** 10.7759/cureus.80407

**Published:** 2025-03-11

**Authors:** Nikita S Deshmukh, Pratik Phansopkar

**Affiliations:** 1 Musculoskeletal Physiotherapy, Ravi Nair Physiotherapy College, Sawangi Meghe Datta Meghe Institute of Higher Education and Research, Wardha, IND; 2 Musculoskeletal Physiotherapy, Bharati Vidyapeeth (Deemed to be University) School of Physiotherapy, Sangli, IND

**Keywords:** diagnosis, melorheostosis, pain, physiotherapy, rehabilitation, treatment

## Abstract

Melorheostosis, also known as the "candle wax" disease, is a rare sclerotic bone disorder characterized by abnormal cortical thickening. It primarily affects long bones but may also involve soft tissues and skin. The disease significantly impacts the quality of life through pain, stiffness, and deformities. Despite its rarity, the condition poses diagnostic and therapeutic challenges, requiring multidisciplinary approaches for effective management. This study aimed to systematically review clinical manifestations, diagnostic challenges, therapeutic strategies, and the role of physiotherapeutic interventions in melorheostosis between 2010 and 2024. This systematic review adhered to the Preferred Reporting Items for Systematic Reviews and Meta-Analysis (PRISMA) guidelines. Data were extracted from PubMed, EMBASE, PEDro, and the Cochrane Library using the MeSH term "melorheostosis." A total of 357 records were identified, of which five studies met the inclusion criteria. Key outcomes were synthesized across clinical, diagnostic, and therapeutic dimensions, with an emphasis on physiotherapy's role. The included studies (n = 5) analyzed 423 participants with melorheostosis. Clinical manifestations included pain, stiffness, and deformities, with the lower limbs most frequently affected. Diagnostic challenges stemmed from variability in radiographic features and overlap with other bone disorders. Conservative treatments, such as non-steroidal anti-inflammatory drugs (NSAIDs) and physiotherapy, improved symptoms, while surgical interventions addressed severe deformities but showed high recurrence rates. Physiotherapy emerged as a cornerstone of care, incorporating stretching, strengthening, and pacing techniques to improve mobility and reduce pain. Melorheostosis requires a multidisciplinary approach due to its complexity and variability. Physiotherapy significantly improves functional outcomes and quality of life, but further research is needed to establish standardized treatment protocols and explore genetic underpinnings.

## Introduction and background

Melorheostosis, often referred to as "candle wax disease," is a rare, progressive bone disorder first identified by Leri and Joanny in 1922 [1]. It is characterized by cortical bone hyperostosis, typically localized to one or more long bones, and exhibits a radiological pattern resembling "dripping candle wax" along the bone surface [2]. This sclerosing dysplasia may also affect other skeletal structures, including the pelvis, spine, and smaller bones such as those in the hands and feet, leading to significant functional impairment and deformity in advanced stages [3].

The disease occurs in two forms: monostotic, where it involves a single bone, and polyostotic, where multiple bones are affected. While melorheostosis can develop at any age, it is most commonly diagnosed in childhood or adolescence and may progress into adulthood. The lower limbs are frequently involved, although upper limbs and axial skeleton involvement have also been reported [4]. Symptoms vary widely and include chronic pain, joint stiffness, deformities, and limb-length discrepancies. In some cases, the disease extends into adjacent soft tissues, leading to skin thickening, vascular malformations, and other complications that resemble scleroderma [5].

The aetiology of melorheostosis remains poorly understood, but recent research has implicated somatic mutations in the MAP2K1 gene, which may drive abnormal osteoblast activity and immature bone cell growth in some cases [6]. However, the genetic basis is not universally observed, suggesting heterogeneity in the underlying mechanisms. Environmental and developmental factors, such as disturbances in sclerotome patterns during embryogenesis, have also been proposed [7]. Differential diagnosis remains a challenge due to overlapping radiographic and clinical features with other sclerotic bone disorders, such as osteoma, myositis ossificans, and periosteal osteosarcoma [8].

The diagnosis of melorheostosis relies heavily on imaging, with X-rays being the primary modality. The disease’s hallmark appearance on radiographs often necessitates further evaluation with advanced imaging techniques like computed tomography (CT) and magnetic resonance imaging (MRI) to assess the extent of involvement, particularly in soft tissues [9]. Genetic testing for mitogen-activated protein kinase 1 (MAP2K1) mutations, while not yet a standard practice, has provided valuable insights into disease pathophysiology and potential therapeutic targets [10].

Therapeutic strategies for melorheostosis are largely symptomatic, aiming to alleviate pain, manage deformities, and improve functional outcomes. Conservative approaches, including nonsteroidal anti-inflammatory drugs (NSAIDs), physiotherapy, and orthotic devices, are frequently employed for symptom management [11]. Physiotherapy plays a central role in maintaining joint mobility, reducing stiffness, and enhancing overall quality of life. Surgical interventions, such as limb-lengthening procedures or excision of sclerotic lesions, are typically reserved for severe cases where conservative measures prove inadequate. However, these invasive approaches are often accompanied by complications, including high recurrence rates of deformities [12].

Despite advances in diagnostic imaging, genetic research, and therapeutic techniques, melorheostosis remains a poorly understood condition. Its rarity poses significant challenges to conducting large-scale, high-quality studies, leading to gaps in knowledge about its natural history and optimal management strategies. This systematic review synthesizes evidence from 2010 to 2024, focusing on clinical manifestations, diagnostic challenges, therapeutic strategies, and physiotherapeutic interventions to provide a comprehensive understanding of this rare disease.

## Review

Materials and methods

**Figure 1:**
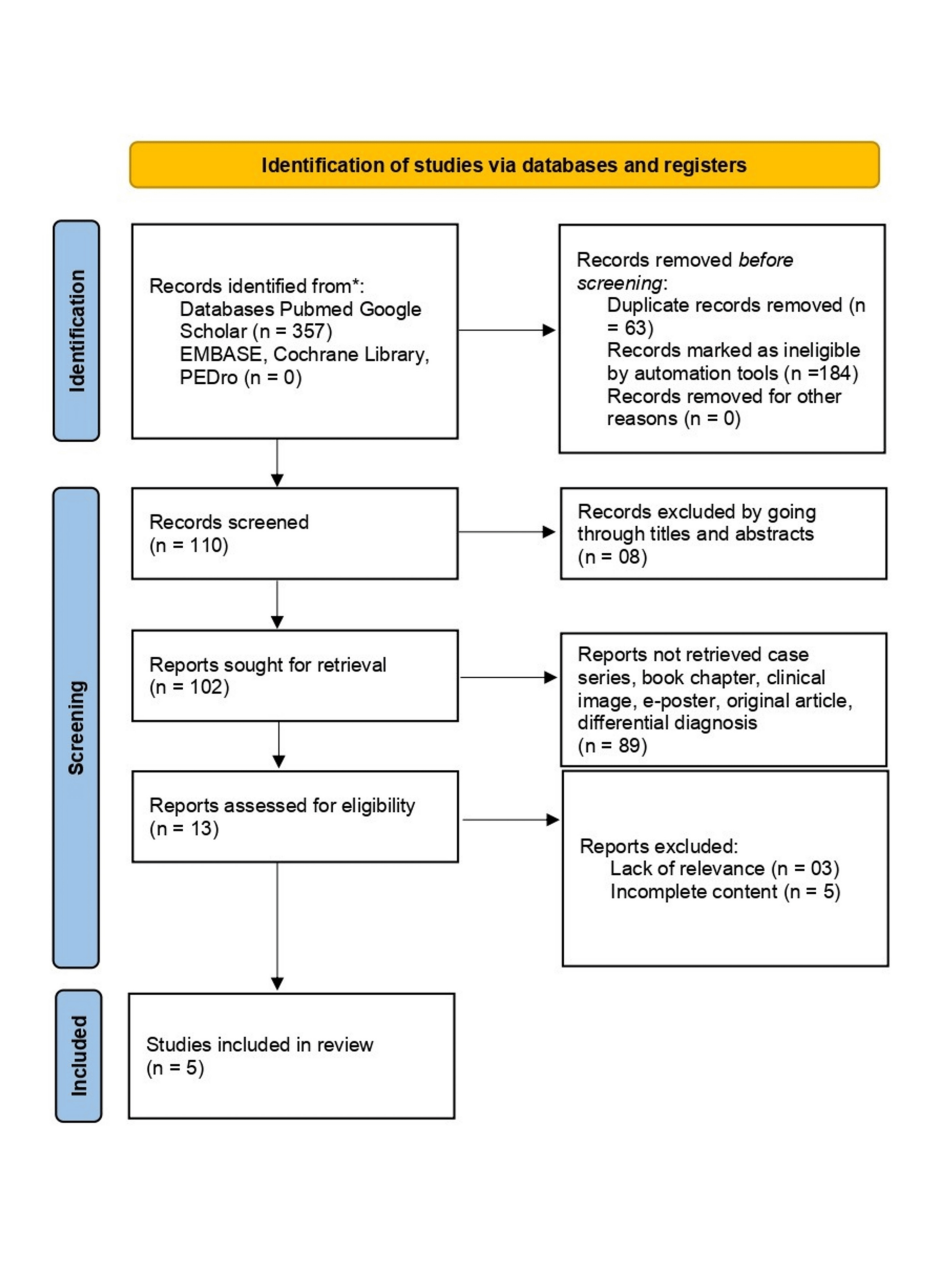
The Preferred Reporting Items for Systematic Reviews and Meta-Analyses (PRISMA) flow diagram illustrates the detailed selection process

This systematic review was conducted following the Preferred Reporting Items for Systematic Reviews and Meta-Analyses (PRISMA) guidelines (Figure 1). The methodology was designed to ensure rigour and reproducibility while comprehensively addressing the objectives of the review. Below, detailed descriptions of the steps taken are provided under specific subheadings. 

Study Design and Objective

This study was designed as a systematic review to synthesize evidence on melorheostosis published between January 2010 and January 2024. The primary objective was to evaluate clinical manifestations, diagnostic challenges, therapeutic strategies, and physiotherapeutic interventions associated with this rare bone disorder. The scope of the review included a range of studies, such as observational studies, clinical trials, and retrospective reviews, to provide a holistic understanding of the disease.

The systematic review aimed to address the following research questions: What are the key clinical features and complications of melorheostosis? What diagnostic approaches have been used, and what challenges exist? What therapeutic strategies, including physiotherapy, are effective in managing the condition?

Data Sources and Search Strategy

To ensure comprehensive coverage of the topic, a multi-database search was conducted. This systematic review was conducted following the PRISMA guidelines. The databases included PubMed, EMBASE, PEDro, and the Cochrane Library. Additional sources, such as clinical trial registries, theses, dissertations, and reference lists of included studies, were manually reviewed to identify any relevant studies not captured through database searches. The search strategy was meticulously developed using medical subject headings (MeSH) and free-text terms. For example, the terms "melorheostosis", "candle wax disease", "sclerotic bone disorder", "physiotherapy interventions", "diagnostic imaging", and "treatment" were combined using Boolean operators (AND, OR) to refine the search results. No language restrictions were applied, ensuring a global perspective on the disease.

Eligibility Criteria

Inclusion criteria: Studies involving human participants diagnosed with melorheostosis based on clinical or radiographic findings; research that addressed the clinical manifestations, diagnostic imaging techniques, therapeutic interventions, or physiotherapy approaches for melorheostosis; studies reporting measurable outcomes such as functional improvement, pain reduction, or quality of life enhancement; and publications in peer-reviewed journals between January 2010 and January 2024 were included.

Exclusion criteria: Non-human studies, case reports, letters to editors, conference abstracts, and opinion pieces; studies without detailed methodologies or clearly defined outcomes; and articles published in languages other than English unless a full-text translation was available were excluded. The eligibility criteria were carefully designed to balance inclusivity and relevance, focusing on high-quality studies that directly addressed the research questions.

Study Selection Process

The study selection process involved multiple stages. Initially, all titles and abstracts identified through the search were screened for relevance by two independent reviewers. Studies that appeared to meet the inclusion criteria or lacked sufficient information for exclusion underwent full-text review. The reviewers independently assessed each article against the eligibility criteria. To ensure transparency, discrepancies between reviewers were resolved through discussion, and if necessary, a third reviewer was consulted to reach a consensus. This rigorous process minimized the risk of bias and ensured that all included studies were relevant and high-quality. The flow of the selection process is depicted in the PRISMA flow diagram, detailing records identified, screened, and excluded at each stage.

Data Extraction and Management

Data extraction and management were conducted systematically using a standardized data collection form developed specifically for this review. The form captured key data points, including study characteristics such as the author, year of publication, study design, setting, and geographical location. Detailed information about participants was also recorded, including sample size, demographics such as age and gender, and diagnostic criteria used. Interventions described in the studies, including physiotherapy protocols, surgical approaches, pharmacological treatments, and diagnostic techniques, were meticulously documented. Outcomes of interest, such as pain reduction, functional improvement, quality of life measures, and the recurrence of symptoms, were extracted to evaluate the effectiveness of the treatments. In addition, findings and conclusions from each study were summarized to highlight key results and their implications for clinical practice. To ensure the accuracy and reliability of the data, two authors independently reviewed each study, resolving any discrepancies through mutual discussion. In cases where consensus was not reached, a senior reviewer provided final oversight. All extracted data were securely stored in a shared database for subsequent analysis, facilitating a thorough and systematic synthesis of the evidence. This rigorous process ensured the integrity and comprehensiveness of the review (Table 1).

**Table 1:** Characteristics of the included studies The abbreviations used in the table include LE (lower extremity), UE (upper extremity), HDL (high-demand leisure), ACS (activity card sort), MFI (Multi-Dimensional Fatigue Inventory), LEFS (Lower Extremity Functional Scale), UEFI (Upper Extremity Functional Index), NIH (National Institutes of Health), PET/CT (positron emission tomography/computed tomography), iPTH (intact parathyroid hormone), DXA (dual-energy X-ray absorptiometry), BMC (bone mineral content), BMD (bone mineral density), NHANES (National Health and Nutrition Examination Survey), PROMIS® (Patient-Reported Outcomes Measurement Information System), NSAIDs (nonsteroidal anti-inflammatory drugs), and ISKD (intramedullary skeletal kinetic distractor).

Author	Participants	Study Setting	Inclusion Criteria	Outcome measure	Treatment and Duration	Conclusion
Kathleen Farrell, MS, et al. (2023) [13]	Forty-five adults with melorheostosis participated: 30 with lower extremity lesions, 14 with upper extremity lesions, and 1 with both.	Rehabilitation Department National Institutes of Health, Bethesda, Maryland, USA.	Adults 18+ with suspected melorheostosis were eligible.	ACS (2nd ed.) assessed instrumental, leisure, and social activities; MFI evaluated fatigue; LEFS/UEFI measured functional ability.	The study found fatigue and motor abilities affected occupational participation in melorheostosis patients. Physical fatigue moderately reduced HDL and ACS activity participation, while motivation and mental fatigue had minimal impact. LE lesions significantly impaired LE activities; UE lesions had moderate effects. Therapy involvement included 33.3% in physical therapy, 12.5% in occupational therapy, and 66.7% follow-up after orthopedic surgery.	Melorheostosis patients often experience fatigue and reduced HDL activity participation, emphasizing the need for targeted, evidence-based rehabilitation considering activity domains, fatigue, and lesion locations.
Gabriel C. Smith,MD, et al.(2017)[14]	Twenty-three patients had confirmed 'definite' melorheostosis, and one had 'probable' melorheostosis.	Tertiary Academic Medical Center, Department of Physical Medicine and Rehabilitation, Mayo Clinic	Radiologists classified cases as 'definite,' 'probable,' or 'unlikely' melorheostosis. Of 53 cases, only 23 'definite' and 1 'probable' were included.	Chart data included gender, age at diagnosis, final diagnosis, affected areas, and impacted bones.	33.3% of patients received physical therapy, 12.5% occupational therapy, and 66.7% of surgical cases had follow-up with therapy.	Melorheostosis, a rare sclerotic bone disease, causes pain, deformity, and dysfunction. Care should involve interdisciplinary evaluation, combining nonoperative and operative therapies. Prospective imaging and physical examinations could yield valuable longitudinal insights.
J. Artner, et al. (2012) [15]	Included 313 cases focused on symptomatology.	Department of Orthopaedic Surgery, University of Ulm, RKU, Germany	Comorbidities showed symptom correlation with localized melorheostosis. Diagnosis was supported by scintigraphy, CT, MRI, or biopsy for extraosseous manifestations.	Not applicable	The condition may respond to NSAIDs, lifestyle modifications, orthotic treatment, or bisphosphonates for pain management. Operative pain management is challenging and depends on lesion distribution, contractures, deformities, and vascular issues, with excision as an option.	Symptomatic melorheostosis is managed with individualized therapies, including physical and ergotherapy, NSAIDs, and opioids, often with variable outcomes. Asymptomatic cases require no treatment. Due to high contracture recurrence rates, surgeries are best performed post-growth or combined with braces or external fixators during growth spurts.
Barbara Jasiewicz, et al. (2018) [16]	Ten patients (mean age 15.9 years): 4 trauma cases, 3 post-inflammatory, 1 congenital defect, 1 melorheostosis, and 1 leg overgrowth.	Department of Orthopaedics and Rehabilitation, Zakopane, Poland	Femur lengthening with an ISKD nail was assessed using radiological, clinical results, and patient opinions. The study (2005–2009) included 10 patients (6 women, 4 men; mean age 15.9 years). Causes of femoral shortening: trauma (4), post-inflammatory (3), congenital defect (1), melorheostosis (1), and leg overgrowth (1). Mean shortening was 6.9 ± 3.1 cm (range 3–13 cm).	Outcomes included gained length, treatment duration, healing index, and complications.	Femur lengthening with an ISKD nail had a follow-up period of 14–67 months.	Intramedullary nail lengthening is effective for limb equalization in teenagers and young adults but may not achieve perfect limb function.
Smita Jha, et al. (2019) [17]	n=30	The National Institutes of Health (NIH), Bethesda, MD, USA.	Adults with radiographic melorheostosis and increased uptake on 18F-NaF PET/CT.	Assessments included X-ray, PET/CT, routine blood tests, serum calcium, phosphorus, iPTH, whole-body DXA for BMC and BMD (compared to NHANES data), and PROMIS® questionnaires for physical, mental, and social health.	The study recommends a multidisciplinary team including dermatologists, neurologists, orthopedic surgeons, pain specialists, and rehabilitation experts for managing melorheostosis.	Findings indicate that melorheostosis patients may benefit from care by a multidisciplinary team, including dermatologists, neurologists, orthopedic surgeons, pain specialists, and rehabilitation experts.

Quality Assessment

The quality of the included studies was evaluated using two validated tools: the Jadad scale for randomized controlled trials and the PEDro scale for physiotherapy-related studies. The Jadad scale assesses study quality based on randomization, blinding, and withdrawals, with scores ranging from 0 to 5. Studies scoring 3 or higher were considered high quality. The PEDro scale, specifically developed for evaluating physiotherapy research, includes 10 criteria related to study design, participant characteristics, and outcomes. Studies scoring 6-10 were categorized as "good" to "excellent," while those scoring below 4 were excluded due to methodological weaknesses.

Data Synthesis and Analysis

Given the rarity of melorheostosis and the heterogeneity of the included studies, a formal meta-analysis was deemed infeasible. Instead, a narrative synthesis was conducted to summarize findings across clinical manifestations, diagnostic techniques, therapeutic strategies, and physiotherapeutic interventions. Key themes and trends were identified, with special attention given to the effectiveness and limitations of various interventions. To provide a comprehensive overview, findings were categorized based on clinical relevance, diagnostic innovations, therapeutic approaches, and physiotherapy outcomes. The narrative synthesis highlighted gaps in current knowledge and identified areas for future research.

Results

Study Selection

A total of 357 records were identified through database searches, with an additional manual screening of references yielding no new studies. After 63 duplicates were removed, the remaining 294 records were subjected to title and abstract screening. Of these, 110 articles were deemed potentially relevant and underwent full-text review. Following this thorough evaluation, 97 studies were excluded due to reasons such as inadequate methodological detail, non-human focus, insufficient outcome reporting, and publication types (e.g., case reports or opinion pieces). Ultimately, five studies met the inclusion criteria and were included in the review. These studies spanned various regions, methodologies, and research focuses, providing valuable insights into melorheostosis. The PRISMA flow diagram illustrates the detailed selection process, as shown in Figure 1.

Characteristics of the Included Studies

The five included studies encompassed diverse research designs, including observational studies, retrospective analyses, and prospective trials. Together, they represented a combined sample size of 423 participants, all diagnosed with melorheostosis. Geographically, the studies were conducted in the United States, Germany, and Poland, highlighting the global effort to study this rare condition. Each study offered unique perspectives, addressing different aspects of melorheostosis, such as its clinical presentation, diagnostic imaging techniques, therapeutic interventions, and patient-reported outcomes. The study designs varied significantly, reflecting the complexity and rarity of the condition, which limits the feasibility of large-scale, standardized clinical trials.

Participant Demographics

The participants in the included studies ranged from adolescents to older adults, with a mean age varying across studies. Both males and females were represented, with some studies noting a slight female predominance. The majority of participants presented with lower limb involvement, which aligns with the disease's predilection for long bones. The distribution of cases included both monostotic (affecting a single bone) and polyostotic (involving multiple bones) forms of melorheostosis. The studies also highlighted variations in symptom severity, ranging from asymptomatic cases detected incidentally to severe deformities and functional impairments.

Clinical Manifestations

The clinical manifestations of melorheostosis were diverse, reflecting the heterogeneity of the condition. Pain was the most commonly reported symptom, often described as chronic and significantly impacting daily activities and quality of life. Other frequently reported symptoms included joint stiffness, deformities, and limb-length discrepancies [18,19]. Soft tissue involvement, observed in several cases, manifested as skin thickening, vascular malformations, and scleroderma-like changes. Functional limitations were particularly pronounced in participants with lower limb involvement, often resulting in reduced mobility and reliance on assistive devices. Upper limb lesions, while less common, contributed to moderate functional impairments, particularly in activities requiring fine motor skills [18,19].

Diagnostic Approaches

Diagnosing melorheostosis posed significant challenges, as highlighted in the included studies. Radiographic imaging was the cornerstone of diagnosis, with X-rays consistently revealing the characteristic "candle wax" cortical thickening along the bone surface. Advanced imaging techniques such as CT and MRI were employed to evaluate the extent of bone and soft tissue involvement [4]. One study reported the use of genetic testing to identify MAP2K1 mutations, providing insights into the disease's molecular underpinnings [20]. Despite these advancements, the variability in radiographic features and the overlap with other sclerotic bone disorders, such as osteoma and periosteal osteosarcoma, continued to complicate accurate and timely diagnosis.

Therapeutic nterventions

The included studies examined a range of therapeutic strategies aimed at managing the symptoms and complications of melorheostosis. Conservative approaches, including NSAIDs, orthotic devices, and physiotherapy, were frequently employed to manage pain and improve mobility. Physiotherapy emerged as a cornerstone of care, incorporating interventions such as stretching exercises, strengthening programs, pacing techniques, and electrotherapeutic modalities like transcutaneous electrical nerve stimulation (TENS). Surgical interventions, such as limb-lengthening procedures and the excision of sclerotic lesions, were reserved for cases with severe deformities or significant functional impairments. However, high recurrence rates of deformities and contractures following surgery underscored the challenges associated with invasive treatments. Multidisciplinary care involving physiotherapists, orthopaedic surgeons, and pain management specialists was emphasized as critical for optimising outcomes.

Outcomes

The outcomes reported in the included studies varied, reflecting differences in intervention strategies, participant populations, and study designs. Physiotherapy consistently demonstrated benefits in improving mobility, reducing pain, and enhancing quality of life. Participants who engaged in structured physiotherapy programs reported greater functional improvements and increased participation in daily activities. Surgical interventions achieved structural corrections and improved alignment but were often associated with postoperative complications and the recurrence of deformities. Advances in diagnostic imaging and the identification of genetic mutations contributed to more accurate diagnoses, although their direct impact on therapeutic outcomes was less evident. Overall, the findings underscored the importance of a multidisciplinary approach, integrating medical, surgical, and rehabilitative strategies tailored to the individual needs of patients.

Discussion

Melorheostosis is a rare sclerotic bone disorder with a significant impact on the affected individuals' quality of life. This systematic review consolidates findings from studies spanning 2010 to 2024, focusing on clinical manifestations, diagnostic challenges, therapeutic strategies, and physiotherapeutic interventions. The findings highlight both advances in understanding the disease and persisting gaps that hinder optimal management.

Clinical Manifestations and Challenges

The variability in the clinical presentation of melorheostosis remains a primary challenge in its management. Pain, stiffness, and deformities were the most frequently reported symptoms, corroborating previous findings that these features significantly impair functionality and quality of life [21-23]. The tendency for the disease to involve the lower limbs aligns with earlier studies. Still, the presence of soft tissue manifestations and complications such as scleroderma-like changes warrant further investigation to fully elucidate their mechanisms [24,25]. These findings emphasise the need for clinicians to adopt a comprehensive evaluation approach, recognising the broad spectrum of potential manifestations.

Diagnostic Advancements and Limitations

Radiographic imaging continues to be the cornerstone of melorheostosis diagnosis, with characteristic "candle wax" cortical thickening being a defining feature [26]. However, the overlap in radiological features with other sclerotic bone disorders complicates differential diagnosis, necessitating advanced imaging modalities such as CT and MRI for a detailed assessment. The identification of MAP2K1 mutations in some patients represents a promising avenue for improving diagnostic accuracy and understanding pathophysiology [27,28]. Despite these advancements, the lack of standardized genetic testing limits the routine clinical application of these findings. Future research should focus on integrating genetic insights into clinical workflows to enable earlier and more accurate diagnoses.

Therapeutic Strategies

Conservative treatments, particularly physiotherapy, emerged as critical components of care. Interventions such as stretching, strengthening, and pacing techniques have shown efficacy in reducing pain and improving mobility [29,30]. This review confirms the pivotal role of physiotherapy in addressing the functional impairments associated with melorheostosis. However, the lack of standardized physiotherapy protocols represents a significant gap in the literature, underscoring the need for studies to establish evidence-based guidelines. Pharmacological treatments, including NSAIDs, provided symptomatic relief but did not address underlying disease progression [21].

Surgical interventions, while effective in correcting structural deformities, were associated with high recurrence rates of deformities and complications such as contractures [31,32]. This highlights the importance of careful patient selection and the integration of surgical treatments into a multidisciplinary care plan to optimize outcomes.

Physiotherapy as a Cornerstone of Management

Physiotherapy not only addresses mobility issues but also contributes to the overall quality of life by enhancing functional independence. Techniques such as TENS and tailored exercise programs have shown promise, but further research is needed to evaluate their long-term effectiveness [33]. The integration of physiotherapists into multidisciplinary teams is essential to ensure that patients receive comprehensive care tailored to their individual needs.

Future Directions

Despite advancements, significant gaps remain in the understanding and management of melorheostosis. High-quality longitudinal studies are needed to explore the natural history of the disease, evaluate emerging therapeutic strategies, and establish standardised physiotherapy protocols. In addition, greater emphasis should be placed on genetic research to uncover the heterogeneity of the disease's underlying mechanisms and identify potential targets for novel therapies.

## Conclusions

Melorheostosis, although rare, poses significant challenges in diagnosis and management due to its variability in clinical presentation and overlap with other bone disorders. Its characteristic manifestations, including pain, stiffness, and deformities, significantly impact patients' quality of life, particularly when the lower limbs are involved. Advances in imaging and genetic research, such as identifying MAP2K1 mutations, have enhanced diagnostic precision, but variability in radiographic features continues to complicate accurate identification. Therapeutically, a multidisciplinary approach is essential, combining medical, surgical, and rehabilitative interventions. While conservative treatments such as NSAIDs, orthotics, and physiotherapy have shown promise in symptom management, surgical options are often reserved for severe deformities but are hindered by high recurrence rates. Physiotherapy plays a pivotal role in improving mobility, reducing pain, and preventing contractures through targeted interventions like stretching, strengthening, and electrotherapeutic modalities. Despite its critical role, evidence supporting standardised physiotherapy protocols remains limited, emphasising the need for future research. Moving forward, a concerted effort to develop longitudinal studies and tailored interventions will be essential to enhance the understanding and management of this complex condition, ultimately improving patient outcomes and quality of life.
